# Chromate Affects Gene Expression and DNA Methylation in Long-Term In Vitro Experiments in A549 Cells

**DOI:** 10.3390/ijms251810129

**Published:** 2024-09-20

**Authors:** Franziska Fischer, Sandra Stößer, Lisa Wegmann, Eva Veh, Tatjana Lumpp, Marlene Parsdorfer, Paul Schumacher, Andrea Hartwig

**Affiliations:** Department of Food Chemistry and Toxicology, Institute of Applied Biosciences (IAB), Karlsruhe Institute of Technology (KIT), 76131 Karlsruhe, Germany

**Keywords:** chromate, epigenetic, DNA methylation, long-term exposure, gene expression profiles, oxidative stress, DNA damage response, inflammation

## Abstract

Chromate has been shown to dysregulate epigenetic mechanisms such as DNA methylation, leading to changes in gene expression and genomic instability. However, most in vitro studies are limited to short incubation periods, although chronic exposure may be more relevant for both environmental and occupational exposure. In this study, human adenocarcinoma A549 cells were treated with 1, 2 or 5 µM chromate for 24 h and compared with incubations with 0.2, 0.5 or 1 µM chromate for 1 to 5 weeks. Chromium accumulated in a pronounced time- and concentration-dependent manner after short-term treatment, whereas a plateau of intracellular chromium content was observed after long-term treatment. While short-term treatment induced a G2 arrest of the cell cycle, this effect was not observed after long-term treatment at lower concentrations. The opposite was observed for global DNA methylation: while short-term treatment showed no effect of chromate, significant dose-dependent hypomethylation was observed in the long-term experiments. Time-dependent effects were also observed in a high-throughput RT-qPCR gene expression analysis, particularly in genes related to the inflammatory response and DNA damage response. Taken together, the results suggest specific differences in toxicity profiles when comparing short-term and long-term exposure to chromate in A549 cells.

## 1. Introduction

Exposure to Cr(VI) compounds has been consistently associated with lung cancer in humans and experimental animals [1]. The oxidation state plays a major role. Only Cr(VI) can pass the plasma membrane via anion channels, while Cr(III) is not readily taken up by cells. However, once inside the cell, Cr(VI) is reduced to Cr(III), which forms stable binary [Cr(III)-DNA] and ternary [ligand-Cr(III)-DNA] DNA adducts in Cr(VI)-treated cells, where the ligand can be ascorbic acid (Asc), glutathione (GSH), cysteine or histidine [2,3]. Perhaps most important, the cellular processing of these lesions has been associated with resistance to mismatch repair and the induction of microsatellite and chromosomal instability [4]. Moreover, in the course of intracellular reduction of Cr(VI) via Cr(V) and Cr(IV) to Cr(III), reactive oxygen species (ROS) are generated, which cause oxidative DNA damage, but which also activate redox-regulated signaling pathways [5,6]. In addition to DNA damage, epigenetic alterations appear to play a role in chromate-induced carcinogenicity. These include changes in DNA methylation, as well as post-translational histone modifications (for a recent review, see [7]).

In general, epigenetic mechanisms are involved in the regulation of gene expression, including the methylation of cytosine to 5-methyl-cytosine, post-translational modifications of various histone residues, and siRNAs [8]. The hypermethylation of DNA in the promoter region prevents the binding of transcription factors and thus leads to the silencing of the respective gene(s) in question [9]. Depending on the actual histone modification, it can lead to a less condensed chromatin structure (euchromatin) with a higher accessibility for transcription factors, or to a tighter packaging (heterochromatin) [10]. The first evidence that chromate affects DNA methylation was observed in Chinese hamster G12 cells, where chromate treatment silenced the *gpt* transgene by increasing DNA methylation [11]. In terms of global DNA methylation patterns, chromate was found to induce hypomethylation in several cancer types and cell culture studies. In the case of post-translational histone modifications, chromate affected the methylation of H3K4, H3K9, H3K27, and H3R2 and the acetylation of H3, H4 and H4K16 [7]. Epigenetic changes have also been observed in lung tumors of chromium workers, showing global hypomethylation, while some tumor suppressor genes were hypermethylated [12,13]. These genes included *APC* [12], *MLH1* [12,14,15], *p16* [12,16], *MGMT*, *HOGG1* and *RAD51* [13]. Furthermore, the reduced expression of MLH1 at the protein level was found in chromate-induced lung cancer and was associated with microsatellite instability [17]. Regarding histone modifications, chromate was shown to decrease *MLH1* mRNA levels, which correlated with the increased dimethylation of H3K9 in the promoter region of the gene [18]. Moreover, it was demonstrated that prolonged, low-dose Cr(VI) exposure resulted in epigenetic dysregulation, related to elevated levels of histone H3 repressive methylation marks (H3K9me2 and H3K27me3) [19].

Regarding gene expression, in a previous study we showed that short-term incubation of A549 and HaCaT cells with higher doses of chromate induced significant changes in gene expression profiles. This included induction of the DNA damage marker *GADD45A*, while specific DNA repair factors were downregulated [20].

While most in vitro studies use short-term exposure conditions with comparatively high concentrations [21], chronic exposure at low concentrations may be more relevant, for both environmental and occupational settings. While direct DNA damage would be expected after short-term exposure, other effects such as epigenetic changes and chromosomal instability may not be detectable under these conditions. Therefore, in the present study, we aimed to investigate the effect of exposure time on several relevant toxicological endpoints at relatively low chromate concentrations in A549 lung cancer cells. We focused on epigenetic changes, as well as on alterations of gene expression profiles and compared higher doses after short-term and lower doses after long-term exposure. Cells were treated with chromate for either 24 h or 1 to 5 weeks; endpoints included cytotoxicity, cellular uptake of chromate, effects on cell cycle distribution and global DNA methylation levels. In addition, a high-throughput RT-qPCR approach was performed to obtain concentration- and time-dependent chromate-induced gene expression profiles related to genomic stability and to identify potential mechanisms of DNA methylation changes.

## 2. Results

To gain a better understanding of chromate-induced long-term effects, particularly with regard to epigenetic mechanisms and gene expression profiles, A549 cells were incubated with non- to moderately cytotoxic concentrations of potassium dichromate for up to 5 weeks. This approach was used to track the time course of the respective toxicological endpoints. Cells were passaged twice a week and incubated continuously with chromate concentrations of 0.2 µM, 0.5 µM and 1 µM after each passaging. To compare long-term and short-term incubation, non- to moderately cytotoxic concentrations of chromate, 1 µM, 2 µM and 5 µM, were selected for 24 h treatment. An untreated control was analyzed at each time point to exclude changes due to prolonged cell cultivation.

### 2.1. Increased Cytotoxicity in A549 Cells after Long-Term Incubation

**Figure 1:**
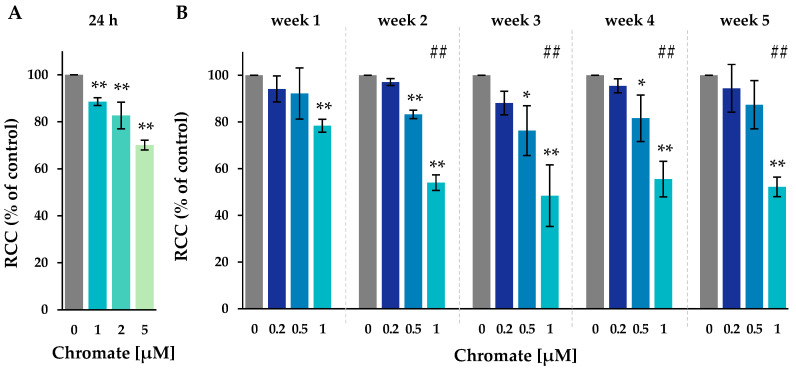
RCC of A549 cells after incubation for 24 h (**A**) or for 1–5 weeks (**B**) with Cr(VI). Shown are mean values of three independent experiments each performed in duplicates ± SD. Statistical analysis was performed to assess differences between treated and untreated cells within a time point using ANOVA followed by Dunnett’s T post hoc test. * *p* ≤ 0.05, ** *p* ≤ 0.01. Differences between 1 µM chromate short-term and long-term experiments were analyzed by ANOVA followed by Dunnett’s T post hoc test. ##: *p* ≤ 0.01.

The cytotoxicity of chromate in A549 cells was determined by calculating the relative cell count (RCC) after 24 h and on a weekly basis between 1 and 5 weeks (Figure 1).

After 24 h, 1 µM chromate caused a slight decrease in cell number to 89%, whereas 2 µM and 5 µM caused a more pronounced decrease to 83% and 70%, respectively. After one week of incubation, the lowest concentration of 0.2 µM chromate showed no change in cell number, indicating no cytotoxicity over the study period. At 0.5 µM, a slight reduction in RCC was observed from week 2 to 83%, which remained at around 80% at subsequent time points. At 1 µM chromate, RCC decreased to 78%, was further reduced to about 50% after two weeks, and remained stable thereafter until week 5. Overall, there was a time- and concentration-dependent increase in Cr(VI)-induced cytotoxicity.

### 2.2. Chromate Uptake in A549 Cells Was Concentration- and Time-Dependent

**Figure 2:**
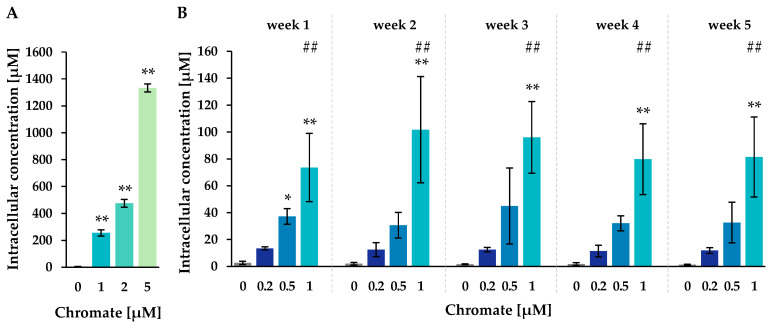
Uptake of chromium in A549 cells after 24 h (**A**) or 1–5 weeks (**B**) of incubation with Cr(VI). The intracellular chromium content was determined via AAS, and intracellular chromium concentrations were calculated. Shown are mean values of three independent experiments, each performed in duplicates ± SD. Statistical analysis was performed to assess differences between treated and untreated cells within a time point by ANOVA followed by Dunnett’s T post hoc test. * *p* ≤ 0.05, ** *p* ≤ 0.01. Differences between 1 µM chromate after short-term and long-term treatment were determined by ANOVA followed by Dunnett’s T post hoc test. ## *p* ≤ 0.01.

To follow the chromate uptake over the period of 5 weeks, the cellular chromium content was measured by atomic absorption spectrometry (AAS). Intracellular concentrations were calculated based on cell number and cell volume (Figure 2).

The untreated control cells showed intracellular chromium concentrations of around 2 µM. After Cr(VI) treatment, a pronounced concentration-dependent accumulation of chromium was observed after both short-term and long-term incubation. The highest intracellular chromium concentration was observed after 24 h at 5 µM, reaching 1333 µM, while 1 µM extracellular chromium resulted in 256 µM. After long-term treatment of 1 to 5 weeks, intracellular concentrations increased almost linearly with higher incubation concentrations, but no further significant time dependence was observed. For example, incubation with 0.2 µM chromate resulted in chromium concentrations between 11.4 µM (week 4) and 13.4 µM (week 1), while incubation with 0.5 µM chromate yielded concentrations between 30.6 µM (week 2) and 44.9 µM (week 3). The highest incubation concentration of 1 µM resulted in intracellular concentrations between 73.6 µM (week 1) and 101.7 µM (week 2). Interestingly, when comparing 1 µM short- and long-term treatment, intracellular concentrations were lower at longer incubation times.

### 2.3. The Cell Cycle Regulation Was Only Affected after Short-Term Treatment at Higher Concentrations

**Figure 3:**
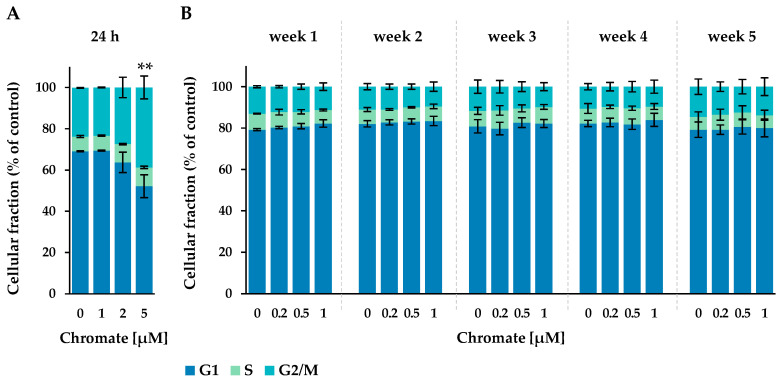
Cell cycle phase distribution of A549 cells after 24 h Cr(VI) incubation (**A**) or incubation for 1–5 weeks (**B**). The cells were stained with DAPI and cell cycle stages were assigned via flow cytometry. Statistical analysis of the G2/M-phase was performed to assess differences between treated and untreated cells within a time point by using ANOVA followed by Dunnett’s T post hoc test. ** *p* ≤ 0.01.

To investigate whether the reduced RCC was due to a cell cycle arrest, the cell cycle distribution was examined by flow cytometry. After treatment, cells were labelled with DAPI staining, measured and assigned to G1, S or G2-/M phase (Figure 3).

After 24 h of incubation, approximately 67% of the cells were in the G1 phase, 8% in the S phase and approximately 25% in the G2 phase. The number of cells in the G2/M phase increased with chromate treatment, starting at 2 µM and reaching 39% at 5 µM, while the proportion in the G1 phase decreased to 52%. This indicates a G2 arrest starting at 2 µM chromate.

In the long-term experiment, the G1 phase accounted for 79% to 84%, the S phase for 6% to 9% and the G2 phase for 10% to 15% of the cells. No time- or concentration-dependent changes were observed under these conditions.

### 2.4. Chromate Induced Hypomethylation of Cytosine Only after Long-Term Treatment

**Figure 4:**
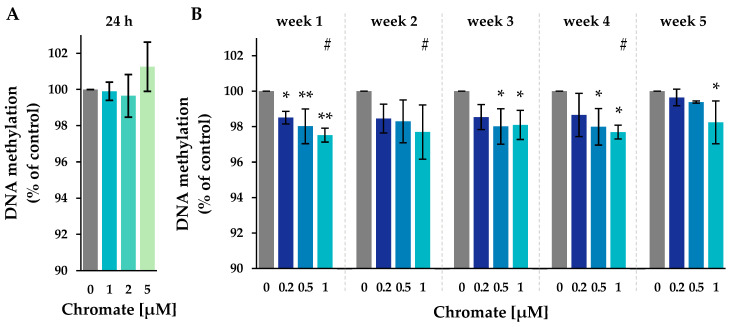
Impact of Cr(VI) on the global 5-methyl-cytosine content in A549 cells after 24 h of treatment (**A**) and after incubation for 1–5 weeks (**B**), shown as a 5-methyl-cytosine level related to the untreated control. DNA isolated from untreated or treated cells was digested into single nucleosides and separated by HPLC on a C18 column. Cytidine was detected at 272 nm and 5-methylcytidine at 280 nm. Shown are mean values of three independent experiments performed in duplicates ± SD. Statistical analysis was performed to assess differences between treated and untreated cells within a time point using ANOVA followed by Dunnett’s T post hoc test. * *p* ≤ 0.05, ** *p* ≤ 0.01. Differences between 1 µM chromate after short-term and long-term treatment were determined by ANOVA followed by Dunnett’s T post hoc test. # *p* ≤ 0.05.

The total amount of 5-methyl-cytosine in DNA was quantified by HPLC-UV analysis. The DNA was cleaved into nucleosides, separated, and quantified by measuring UV absorption. The 5-methyl-cytosine content was related to the total cytosine content (unmethylated cytosine + 5-methyl-cytosine). Relative levels are shown in Figure 4; absolute 5-methyl-cytosine levels are shown in Supplementary Figure S1.

The level of 5-methyl-cytosine in the untreated control ranged from 3.15% to 3.17% throughout the study (see Supplementary Figure S1). After 24 h of incubation, the methylation level remained unchanged at all chromate concentrations investigated. In the long-term experiments, methylation decreased significantly, with the most pronounced reduction occurring after week 1. No further decrease was observed until week 5. The decrease in methylation was dose-dependent, reaching 97.7% at 1 µM.

### 2.5. Impact of Chromate on Gene Expression Profiles

The effect of chromate on the gene expression profile of 95 selected genes for 24 h and over a period of 5 weeks was investigated using high-throughput RT-qPCR. The gene set included genes encoding proteins involved in metal homeostasis, epigenetics, inflammation, oxidative stress response, DNA damage response, apoptosis, and cell cycle regulation, as described previously [16,22]. A complete list of genes and their encoded proteins is provided in the Supplementary Table S1. The relative gene expression was calculated by normalizing treated samples to the untreated control and expressed as log_2_ values. Reductions of at least 50% (log_2_-fold change ≤ −1) or twofold inductions (log_2_-fold change ≥ 1) compared to the untreated control were considered relevant, and concentration-dependent trends were also considered. For genes related to epigenetic regulation, the gene set included those encoding enzymes involved in DNA methylation, DNMT1, DNMT3a and DNMT3b, and those encoding enzymes involved in DNA demethylation, TET1, TET2 and TET3. Genes encoding selected enzymes involved in histone modification were also considered. This includes, among others, the acetyltransferase Ep300, the methyltransferases EHMT2 and SETD2, the deacetylases HDAC1, HDAC2, HDAC3 and HDAC10, as well as the demethylase KDM3A.

**Figure 5:**
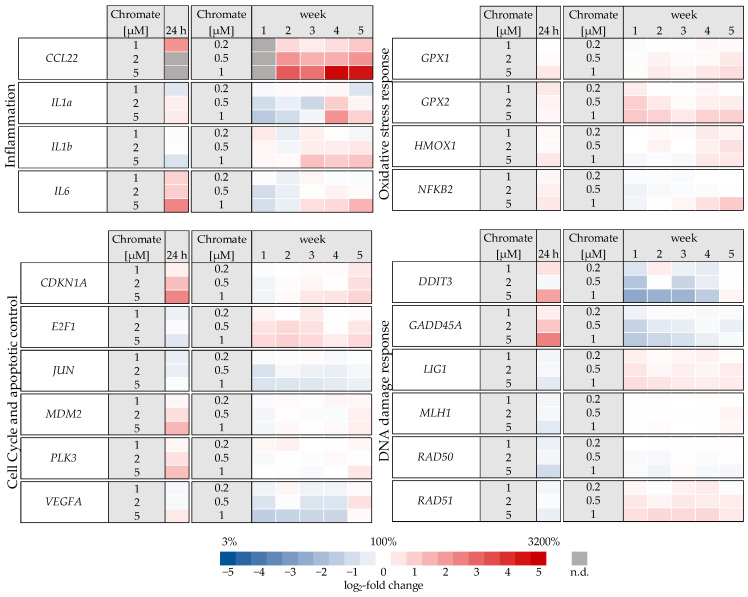
Overview of gene expression profiles of A549 cells treated with Cr(VI) for 24 h or 1–5 weeks using a high-throughput RT-qPCR. Genes depicted encode proteins involved in apoptotic and cell cycle control, inflammation, DNA damage response, and oxidative stress response. The log_2_ changes relative to the untreated control are illustrated. Blue represents a repression, and red represents an induction. Shown are mean values of at least three independent determinations performed in duplicates. n.d. = not determinable.

Overall, changes in the expression of genes related to epigenetic regulation were minimal, and no effects considered relevant were identified, either after 24 h or after long-term treatment (Supplementary Figure S2). In contrast, more pronounced effects were observed on selected genes related to other clusters mentioned above (Figure 5). A comprehensive summary of the effects of chromate on all genes investigated is presented in the Supplementary Materials (Figures S2–S4).

**Figure 6:**
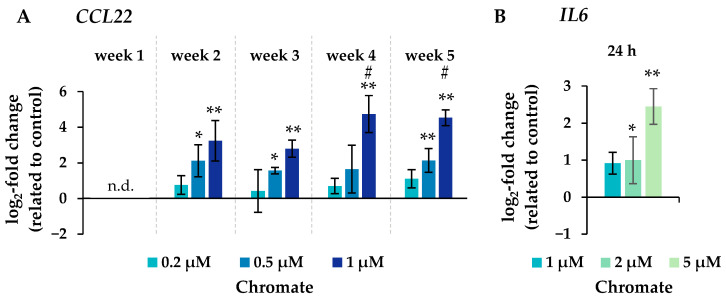
Changes in the expression of the inflammatory genes *CCL22* after incubation for 1–5 weeks (**A**) and *IL6* after 24 h of treatment (**B**) with chromate in A549 cells. Shown are mean values of at least three independent experiments performed in duplicates. Statistical analysis was performed to assess differences between treated and untreated cells within a time point followed by Dunnett’s T post hoc test. * *p* ≤ 0.05, ** *p* ≤ 0.01. Differences between the short-term (24 h) and long-term (1 to 5 weeks) experiments were determined by ANOVA followed by Dunnett’s T post hoc test. # *p* ≤ 0.05. n.d. = not determinable.

Significant changes in gene expression were observed, particularly in relation to the inflammatory response, cell cycle control, DNA damage response and, to a lesser extent, oxidative stress response. Regarding the inflammatory response, *CCL22* in particular was strongly induced after 24 h incubation, reaching 4-fold higher expression levels compared to the control (Figure 6). Even stronger effects were observed after long-term incubation, both concentration- and time-dependent, starting at 0.2 µM chromate and reaching a 26-fold induction after 5 weeks at 1 µM chromate. *IL6* was clearly induced in a dose-dependent manner after 24 h, but the effects were much less pronounced after long-term incubation. *IL1a* and *IL1b* were not induced after short-term incubation but were upregulated after long-term treatment, with the strongest effects after 3 and 4 weeks, respectively.

**Figure 7:**
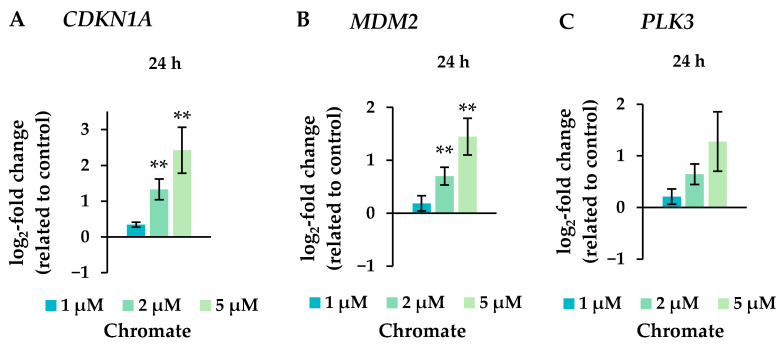
Expression levels of the cell cycle and apoptotic control genes *CDKN1A* (**A**), *MDM2* (**B**), and *PLK3* (**C**) in A549 cells after 24 h treatment with Cr(VI). The log_2_ changes relative to the untreated control are illustrated. Shown are mean values of at least three independent determinations performed in duplicates. Statistical analysis was performed to assess differences between treated and untreated cells by ANOVA followed by Dunnett’s T post hoc test. ** *p* ≤ 0.01.

**Figure 8:**
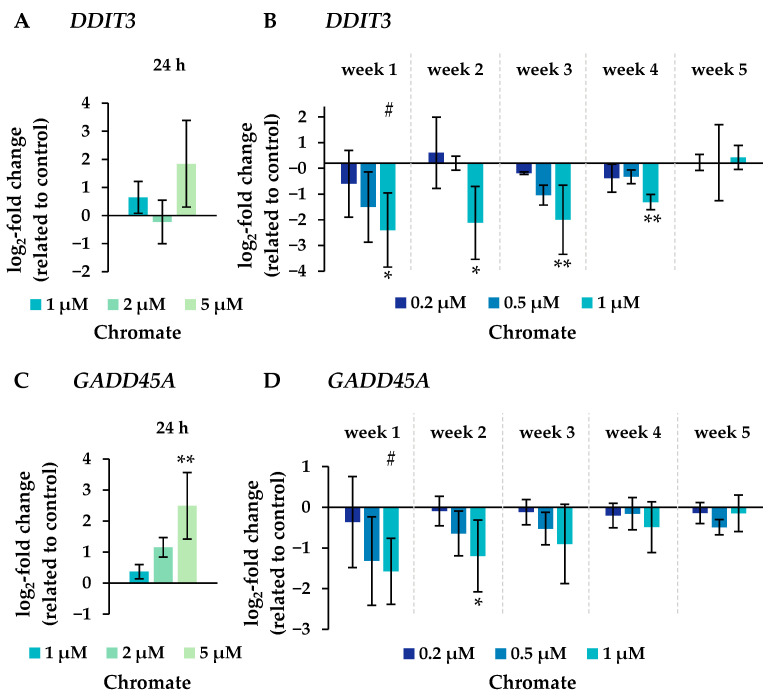
Expression levels of genes related to DNA damage response *DDIT3* (**A**,**B**) and *GADD45A* (**C**,**D**) in A549 cells after 24 h treatment (**A**,**C**) and after incubation for 1–5 weeks (**B**,**D**) with Cr(VI). The log_2_ changes relative to the untreated control are illustrated. Shown are mean values of at least three independent determinations performed in duplicates. Statistical analysis was performed to assess differences between treated and untreated cells within a time point using ANOVA followed by Dunnett’s T post hoc test. * *p* ≤ 0.05, ** *p* ≤ 0.01. Differences between short-term and long-term trials were determined by ANOVA followed by Dunnett’s T post hoc test. # *p* ≤ 0.05.

Several oxidative stress response genes were included, namely *GPX1*, *GPX2*, *HMOX1* and *NFKB2.* Only small effects were observed, most of which were not considered relevant after short-term treatment. Nevertheless, slight upregulations were observed after 4 and 5 weeks of treatment at the highest concentration and in the case of *GPX2* also starting after treatment with 0.5 µM chromate after 1 week and longer. For N*FKB2*, a 2-fold induction was observed after 5 weeks of treatment. Some cell cycle and apoptosis-related genes such as *CDKN1A*, *E2F1*, *MDM2*, *PLK3* and *VEGFA* were also affected by chromate. Within this cluster, there were again clear differences between short- and long-term treatment. The most pronounced effects were observed for *CDKN1A*, *MDM2* and *PLK3* (see Figure 7 for details). *CDKN1A* was upregulated by chromate already at short-term treatment, starting at 2 µM. With long-term treatment, an upregulation was seen only after 5 weeks and was much less pronounced. *PLK3* and *MDM2* were induced after 24 h starting from 2 µM, whereas these genes were hardly affected in the long-term experiment. In contrast, *VEGFA* was slightly downregulated after week 1 and thereafter at 1 µM chromate. Genes associated with damage signaling, *DDIT3* and *GADD45A*, were upregulated after short-term treatment (see Figure 8); in the case of *GADD45A*, the induction was dose-dependent and significant, reaching more than 4-fold levels at 5 µM chromate. In contrast, both genes were downregulated after long-term treatment, in the case of *GADD45A* to about 34% and in the case of *DDIT3* to about 17% after week 1. In both cases, this repression decreased with increasing treatment duration, and gene expression returned to control levels by week 5. Interestingly, genes encoding specific DNA repair factors such as *LIG1*, *MLH1*, *RAD50*, and *RAD51* were not affected after 24 h, except for a slight downregulation of *RAD50* at 5 µM chromate, and a tendency towards induction in the case of *LIG1* and *RAD51* in the long-term experiments.

## 3. Discussion

In this study, we observed clear differences in the toxicity profile of chromate between short-term (24 h) and long-term (up to five weeks) treatment of A549 cells. Concentrations were chosen to cover the range from no to moderate toxicity and to include an overlap of concentrations between short-term and long-term treatment. This resulted in up to 5 µM chromate for 24 h and 0.2 to 1 µM chromate for long-term treatment, respectively. Special focus was given to epigenetic changes, on the level of both DNA methylation and gene expression. In addition, cells were analyzed for chromate uptake and intracellular accumulation, cell cycle progression and gene expression, focusing on oxidative stress, cell cycle regulation, inflammation and DNA damage response.

RCC was used as a marker of cytotoxicity. The toxicity of Cr(VI) is attributed to its uptake through plasma membrane anion channels and intracellular reduction to Cr(III). During reduction, highly reactive chromium intermediates Cr(V) and Cr(IV) are generated, as well as ROS, which facilitate the oxidation of a variety of molecular targets, such as phospholipids, proteins, and DNA [2,3]. As expected, a concentration- and time-dependent increase in cytotoxicity was observed, consistent with previous studies in various cell lines reported in the literature that investigated Cr(VI) toxicity for up to five days [23,24,25]. In the present study, cytotoxicity was more pronounced in the long-term experiments than after 24 h and remained fairly constant after 2 weeks. Uptake studies revealed a pronounced concentration-dependent intracellular accumulation, resulting in up to 280-fold higher intracellular chromium concentrations compared to extracellular chromate concentrations. This accumulation is explained by the intracellular reduction of Cr(III) and binding to cellular macromolecules as described above. As a result, chromium is trapped intracellularly in the form of Cr(III), presumably leading to its extensive accumulation. Similar effects have been described previously in the literature [26,27,28]. Interestingly, significantly higher chromium levels were detected after 24 h when compared to long-term incubation over several weeks. Furthermore, in the long-term experiment, no time dependence could be detected for any concentration tested, and the intracellular levels remained constant over the five weeks studied. The short-term data confirm that water-soluble chromate is rapidly absorbed within a few hours, as demonstrated previously for A549 cells [29,30], as well as for up to 72 h in human lung fibroblasts [26]. Taken together, these data suggest that chromate uptake occurs in a time-dependent manner immediately after incubation, with saturation observed after long-term treatment. The reason for the lower intracellular concentrations after long-term treatment remain unclear. It may be due to a slightly higher degree of cell confluency compared to short-term treatment. Furthermore, the chromium content may be diluted via cell division under these conditions. In any case, it can be assumed that time-dependent effects such as increased cytotoxicity or epigenetic changes are due to the prolonged exposure and not to differences in intracellular chromium content.

Cytotoxicity, measured as a reduction in cell growth, could be due to cell death or the induction of cell cycle arrest. Therefore, the cell cycle distribution and progression were investigated. The cell cycle profile measured in the present study is consistent with previous studies in A549 cells as described in the literature [31,32,33]. In the absence of chromate, a higher proportion of cells in the long-term experiments were in the G1 phase compared to the cells in the short-term experiment. This may be due to a slightly higher density and therefore higher confluence, as discussed above for the uptake studies. However, differences in confluency are unlikely to affect the effect of chromate on cell cycle distribution because untreated controls were included at each time point to which the chromate-induced effect was related. Upon treatment with chromate, there were pronounced time-dependent differences. While the short-term treatment affected the cell cycle distribution and induced a G2/M arrest, after long-term treatment, there was no change in the cell cycle distribution compared to the untreated control. Gene expression profiles related to cell cycle control and apoptosis showed an induction of *CDKN1A*, which encodes p21, after 24 h, whereas no changes in the expression of cell cycle related genes were observed after long-term treatment. However, it should be noted that *CDKN1A* was only induced at 2 and 5 µM chromate, whereas in the long-term experiments, only concentrations up to 1 µM chromate were used. An impact of Cr(VI) on cell cycle arrest has also been described in the literature for various cell lines [31,32,34]. While Xie et al. observed a G2 arrest in WTHBF-6 cells after 24 h incubation starting at 2.5 µM chromate [35], Lou et al. found a G1 arrest in A549 cells after 24 h incubation starting from 10 µM [31]. A slight G2 arrest was also described by Zhang et al. in A549 starting at 1 µM chromate [32]. One aspect may be the use of normal vs. cancer-derived human lung cell lines, but even for the same cell line, A549, different results have been reported. No literature data are available on the long-term exposure conditions used in this study.

Chromium-induced carcinogenesis is associated with changes in global and gene-specific methylation levels. Global hypomethylation caused by chromate has already been demonstrated in vivo upon 4 weeks of oral exposure via drinking water [36] and in vitro [27]. However, while most in vitro studies investigating the effects of chromate on DNA methylation were limited to short incubation periods of up to 48 h [13,14,27,37], in the present study, we demonstrated that effects were not evident within 24 h of short-term incubation but required long-term treatment. Thus, global cytosine methylation was reduced in a concentration-dependent manner over a total exposure period of 5 weeks. Global hypomethylation has been associated with genomic instability and with cancer [38]. In general, this could be due to reduced DNA methylation or the induction of DNA demethylation enzymes. To investigate whether changes in gene expression were involved in the case of chromate, gene expression profiles of enzymes involved in DNA methylation were generated. However, the expression patterns differed only slightly from the control, indicating that neither the induction nor the repression of TET enzymes or DNMTs were involved in the observed hypometabolism. This suggests that changes are likely to be due to interactions at the protein level. For example, the activity of DNMTs could be affected, which are involved in transferring the methyl group to the daughter strand during cell division [39]. This effect would require several cell divisions to manifest itself, which would explain the restriction to long incubation times. In support of this theory, it has been shown that mouse hepatoma Hepa-1c1c7 (Hepa-1) cells treated with chromate caused the cross-linking of DNMT1 with HDAC1, which resulted in reduced methylation activity; this may be attributed to Cr(III) formed upon the intracellular reduction of Cr(VI) [40]. Furthermore, the global hypomethylation induced by chromate in vivo was correlated with elevated levels of malondialdehyde (MDA), an indicator of oxidative stress [36]. Although the induction of oxidative stress-related genes was rather weak in the present study, it should be noted that *Nrf2* is constitutively overexpressed in A549 cells [41], while it is suppressed in other cell lines under basal conditions. This may lead to an altered oxidative stress response in A549 cells. The mechanism by which oxidative stress may lead to hypomethylation is not yet fully understood; potential mechanisms include increased levels of DNA lesions, as well as interaction with the cofactor S-adenosyl-methionine (SAM) [42,43]. In addition, chromium may interact with critical structures within enzymes involved in the regulation of epigenetic modifications. Thus, CXXC zinc finger structures are components of several DNA methyltransferases, including DNMT1. These structures are characterized by the presence of conserved cysteine residues complexed by zinc, which are critical for the recognition and binding of specific DNA sequences. In the case of DNMT1, the binding of the CXXC zinc finger domain to DNA regions rich in cytosine and guanine (CpG) is essential for its catalytic activity [44,45,46]. To the best of our knowledge, there are no literature data on the direct interaction of chromate with zinc-binding proteins involved in histone modifications. More information is available, for example, in the case of arsenite, which was found to bind directly to the cysteine-rich zinc-binding ADD domain of DNMT3A. This enzyme plays a crucial role in the establishment of new DNA methylation patterns during development and DNA repair processes [47]. Additionally, due to the complexation of zinc to cysteines within zinc-binding structures, they may also be particularly susceptible to oxidative stress induced by chromate. Thus, one study used a thiolate complex to simulate a 2-cysteine, 2-histidine zinc finger; the results indicated the generation of disulfides and Cr(III)-thiolate complexes upon chromate treatment [48]. DNA methylation is also closely linked to changes in post-translational histone modifications. Several studies have shown that chromate affects different histone modification patterns [18,19,49,50,51]. In our study, we analyzed the gene expression encoding several histone modifications regulating enzymes using high-throughput RT-qPCR. However, the expression of the analyzed genes was not significantly altered by chromate treatment. At the protein level, several histone modifying enzymes have been shown to be affected by chromate, such as EHMT2 G9a [18,19], as well as HDAC2 and HDAC3 [50].

We also investigated DNA damage and the inflammatory response at the transcriptional level. In particular, in the area of inflammation, the induction of the interleukin genes *IL1a*, *IL1b* and *IL6*, as well as *CCL22*, a gene encoding a macrophage-derived chemokine, were observed. This is consistent with a chromate-induced inflammatory response in cells, as described in the literature [52]. A possible signaling pathway of the inflammatory response is via NFκB, regulating the expression of *IL1b*, *IL6* and *NFKB2.* The involvement of NFκB in chromate-induced inflammation was previously demonstrated in Beas-2B cells after 6 months of incubation [53]. The authors also showed cytokine secretion, which supports the gene expression results of our experiments. Meanwhile, *CCL22*, which was surprisingly most induced of all the genes investigated within this study, can be activated by various stimuli, including interleukin-1 [54].

One important mode of action of Cr(VI) is to induce DNA damage after intracellular reduction. In addition to the formation of highly reactive Cr(V) and Cr(IV) intermediates, which may lead to the generation of ROS, stable binary (Cr(III)-DNA) and especially ternary Cr(III)-DNA adducts are formed involving cellular reducing agents such as ascorbate or glutathione [3,7,55]. Regarding the DNA damage response in the present study, there was a pronounced dose-dependent induction of *DDIT3* (DNA-damage-inducible transcript 3) and *GADD45A* (growth arrest and DNA damage inducible gene α) after 24 h of incubation, in agreement with our previous study [20]. *DDIT3* is involved in DNA damage signaling, but the encoded protein is also part of a pro-apoptotic signaling pathway [56,57], and *GADD45* is also upregulated by genotoxic stress [58]. Interestingly, both genes were downregulated after long-term treatment between weeks 1 and 4 and returned to control levels at week 5. At first sight, the repression of these stress sensors does not seem to be beneficial for the cell. However, it has already been shown that *DDIT3* [59] and *GADD45A* [60] are repressed by NFκB in cancer cells to ensure cell survival and may therefore be important for adaptation. Regarding genes encoding specific DNA repair factors, no significant changes were observed after either short-term or long-term treatment, except for a slight downregulation of *RAD50* after 24 h at 5 µM chromate and a slight induction of *RAD51* after long-term exposure to 1 µM chromate. This seems to contradict previous results, where genes encoding DNA repair proteins across all major DNA repair pathways were consistently downregulated by chromate in the same cell line [20]. However, much higher chromate concentrations were used in the latter study, and downregulation was only observed at 6.6 µM chromate and above. Interestingly, DNA double-strand break (DSB) repair plays an important role in the cellular response to chromate. While acute exposure to chromate provoked DSBs and induction of homologous recombination (HR) prolonged exposure to Cr(VI) inhibited HR and RAD51 nucleofilament formation due to decreased nuclear RAD51 protein levels and corresponding mRNA levels [23,61]. Thus, decreased DSB repair may be an important mechanism in chromate-induced chromosomal instability and adaptation to tumor development.

## 4. Material and Methods

### 4.1. Materials

All chemicals were purchased from Sigma-Aldrich (Taufkirchen, Germany) or Carl Roth (Karlsruhe, Germany). Potassium dichromate (≥99.5%) was obtained from Carl Roth (Karlsruhe, Germany). Cell culture medium and additives were obtained from Sarstedt (Nuembrecht, Germany,) and fetal bovine serum (FBS) was obtained from Thermo Fisher Scientific (Dreieich, Germany). All cell culture materials were bought from Sarstedt (Nuembrecht, Germany), and PCR consumables were purchased from Brand (Wertheim, Germany). Chromium AAS standard (1 g/L) was obtained from Carl Roth (Karlsruhe, Germany). FACSFlow and FACSRinse were bought from BD (Heidelberg, Germany), DAPI (1 g/L) was bought from Sigma-Aldrich (Taufkirchen, Germany), primers for RTqPCR were synthesized by Eurofins (Ebersberg, Germany). PCR reagents were obtained from Machery-Nagel (Dueren, Germany), Applied Biosystems (Forster City, WI, USA), Fluidigm (San Francisco, CA, USA), Bio-Rad (Munich, Germany), New England Biolabs (Frankfurt am Main, Germany), and Teknova (Hollister, CA, USA).

### 4.2. Cell Culture and Exposure to Chromate

For all experiments, A549 cells (ATCC CCL-185), a human adenocarcinoma cell line, were used. Before the beginning of the experiments, an STR analysis was conducted according to ICLAC (International Cell Line Authentication Committee), confirming a matching coefficient of 100% related to the original cell line. The cell line has been tested to be free of mycoplasms. Cells were grown in RPMI-1640 cell culture medium with the supplement of 10% fetal calf serum (FBS) and 100 U/mL penicillin and 100 µg/mL streptomycin (complete medium) at 37 °C and an atmosphere with 5% CO_2_. Cells were cultivated to a confluence of 80–90%. Only cells of passages 14–30 were used for the experiments. Logarithmically growing cells were incubated with K_2_Cr_2_O_7_ in the short-term experiments for 24 h with concentrations of 1 µM, 2 µM and 5 µM chromate, and in the long-term experiments for 1–5 weeks with 0.2 µM, 0.5 µM and 1 µm chromate. For the long-term experiments, the cells were passaged every 3–4 days and re-incubated with new chromate-containing complete medium. The non-exposed control cells were concurrently cultivated and sub-cultured over the same period to discern the effects of continuous passaging from those of sustained exposure to chromate.

### 4.3. Cellular Uptake

To determine the cellular chromium uptake, the chromium content was measured by GF-AAS (PinAAcle 900 T, Perkin Elmer, Rodgau, Germany). The measurements were calibrated with standard chromium solutions. The limit of detection was quantified as 0.079 µg/L, the limit of quantification as 0.158 µg/L and the limit of determination as 0.291 µg/L. Each value was derived from three independent experiments, each performed in duplicates and measured in three technical replicates, with standard deviations of ≤5%. Intracellular concentrations were calculated based on the cell number and average cell volume, both determined via the CASY^®^ TT cell counter (OMNI Life Science, Bremen, Germany). Subsequently, the cells were pelleted, dissolved in 500 µL of a 1:1 mixture of 30% H_2_O_2_ and 69% HNO_3_ (*v*:*v*) and heated stepwise up to 95 °C. The residue was dissolved in 1 mL of 0.2% HNO_3_ and measured and the chromium content was calculated using an external calibration. The following temperature program was used: drying at 120 °C for 30 s and 140 °C for 45 s, pyrolysis at 1500 °C for 30 s, atomization at 2300 °C for 5 s, and cleaning at 2450 °C for 3 s.

### 4.4. Determination of Cell Cycle Distribution

The cell cycle distribution was determined by flow cytometry (BD, Heidelberg, Germany). About 10^6^ cells were dissolved in 1 mL of PBS and fixed with 3 mL of 80% ethanol (−20 °C). The cells were stored at −20 °C overnight. Cells were then pelleted, washed with PBS and stained with DAPI solution (Partec, Münster, Germany). Fluorescence was measured using a BD LSRFortessa flow cytometer (BD, Heidelberg, Germany) with a violet laser at an excitation wavelength of 405 nm and a bandpass filter of 450/50 nm. To assess the distribution of the cell cycle, the fluorescence signal was graphed against the cell number in a histogram.

### 4.5. Measurement of Global DNA Methylation

In order to measure the 5-methyl-cytosine content, the cells were pelleted and the DNA was isolated using the Monarch gDNA purification kit (New England Biolabs, Frankfurt am Main, Germany). The purity and amount of DNA was then determined by measuring the absorbance at 260/230 nm with the TECAN Infinite m200 Pro. For the measurement of the global 5-methyl-cytosine content, DNA was digested with a nucleoside digestion mix (New England Biolabs, Frankfurt am Main, Germany) and nucleosides were separated by high performance liquid chromatography (HPLC) (Thermo Fisher Scientific, Dreieich, Germany) on a Luna 5u C18(2) 100A column (Phenomenex, Aschaffenburg, Germany). Milli-Q water, a 50 mM sodium acetate buffer (pH4), and methanol were used as eluent at a flow rate of 1 mL/min. The composition is shown in Table 1. The content of cytidine and 5-methyl-cytidine was measured by UV absorbance at 272 nm for cytidine and 280 nm 5-methyl-cytidine, respectively, and calculated by external calibration. The calculation was performed according to the following formula:

$$\mathrm{Genomic}\;5-\mathrm{methyl}-\mathrm{cytosine}\;\left(\%\right)=\frac{n\left(\;5-\mathrm{methyl}-\mathrm{cytidines}\right)}{n\;\left(5-\mathrm{methyl}-\mathrm{cytidines}\right)+n\;\left(\mathrm{cytidines}\right)}$$

### 4.6. Gene Expression Analysis Using High-Throughput RT-qPCR

Gene expression analysis was performed as described previously [62,63]. PCR was carried out in a 96 × 96 Dynamic Array integrated fluidic circuit (Fluidigm, San Francisco, CA, USA), enabling the parallel analysis of 96 samples on the expression of 95 genes. In addition to genes related to metal homeostasis, oxidative stress response and inflammation, the gene set also included 16 genes of epigenetic regulation, as described in [22], and is listed together with the encoded proteins in Table S1 in the Supplementary Materials. For analysis, cells were pelleted and RNA was isolated using NucleoSpin RNA Plus kit (Machery-Nagel, Dueren, Germany). The measurement of RNA content and purity was carried out by applying the TECAN Infinite M200 Pro. Transcription into cDNA was performed using qSkript cDNA Synthesis kit (QuantaBio, Beverly, MA, USA), and preamplification was conducted using a pooled primer mix and the TaqMan PreAmp Master Mix (Applied Biosystems, Darmstadt, Germany). Immediately afterwards, exonuclease digestion was performed using Exonuclease I (Thermo Fisher Scientific, Dreieich, Germany). The quality control was performed as described previously [62] by including the corresponding controls on each chip. Thus, the combination of the reactionless control (NRC) without primers, the NTC and NTC-STA without cDNA, and the NoRT control enabled the detection of potential impurities in the reagents, as well as reactions leading to the formation of unintended targets, primer dimers and gDNA background. The data processing was performed using the GenEx software, version 5.3.6.170. For normalization, up to 5 reference genes (*ACTB*, *B2M*, *GAPDH*, *GUSB*, and *HPRT1*) were employed. To determine the optimal combination of reference genes for analysis, two integrated programs, geNorm and Normfinder, were applied. GeNorm compares the relative expression of gene pairs across different samples and sequentially eliminates the gene with the highest expression variation. This process identifies the most suitable gene pair for normalization. Normfinder employs a specialized variance analysis to compute an average expression for all genes, which is then compared to the individual expression of each gene, resulting in a standard deviation. This approach additionally considers various sample treatments, enabling the identification of unstable or regulated genes that are subsequently excluded as reference genes. The application of both programs led to the identification of the most appropriate reference genes for each analysis. Subsequently, normalization was performed using these selected genes. The ΔΔCq method was used for semiquantitative analysis, relating treated samples to the untreated control.

### 4.7. Statistical Analysis

Cell culture studies were performed in at least 3 independent experiments, each in duplicate determination with two parallel treatments, resulting in 6 determinations for all experiments. For gene expression, 2 additional technical replicates were analyzed. Data are expressed as mean ± standard deviation (SD). Differences between untreated and chromate treated samples as well as between different time points were analyzed by one-way ANOVA following Dunnett’s post hoc test. Statistical significance was tested using *p*-values ≤ 0.05 and ≤0.01. Analysis was performed using Real Statistics Resource Pack software (version 7.3.2), copyright (2013–2021) Charles Zaiontz (www.real-statistics.com accessed on 7 September 2021).

## 5. Conclusions

The present study investigated the effects of short-term (24 h) and long-term (1 to 5 weeks) exposure to chromate in A549 cells. Cytotoxicity, intracellular chromium accumulation, global DNA methylation, gene expression profiles and cell cycle regulation were compared. The results show pronounced differences between short-term and long-term treatment, providing insight into time-dependent changes in cellular response. Chromate uptake was rapid and led to pronounced intracellular accumulation. Effects on cell cycle progression were observed only after 24 h and were limited to higher concentrations, as also reflected in the gene expression profiles. DNA hypomethylation, on the other hand, required the long-term exposure of at least 1 week, with no further changes up to 5 weeks. At this endpoint, significant effects were observed at the lowest concentration of 0.2 µM chromate. Changes at the transcriptional level could be excluded as the underlying mechanism, since no changes in gene expression were observed in any of the genes encoding enzymes related to epigenetic modifications. Therefore, interactions at the protein level may be more relevant; for example, the zinc-binding structures of DNA methyltransferases may represent potentially sensitive targets. Time-dependent effects were also observed in the inflammatory and DNA damage response. Overall, the long-term in vitro experiments conducted in the present investigation provide valuable insights into immediate vs. persistent cellular responses to chromate, such as epigenetic changes, and may better reflect effects relevant to chronic exposure.

## Figures and Tables

**Table 1:** Composition of the eluate for the separation of nucleosides by HPLC.

Retention Time [min]	A	B	C
0–6.6	77%	15%	8%
6.7–16.6	72%	15%	13%
16.7–25	77%	15%	8%

A: ddH_2_O, B: 50 mM sodium acetate buffer (pH 4), C: methanol.

## Data Availability

The data presented in this study are available on request from the first (F.F.) and corresponding author (A.H.) for researchers at academic institutes who meet the criteria for access to the confidential data.

## Supplementary Materials

The supporting information can be downloaded at https://www.mdpi.com/article/10.3390/ijms251810129/s1.
