# Supplementary material for: Chromate Affects Gene Expression and DNA Methylation in Long-Term In Vitro Experiments in A549 Cells
Source: Int J Mol Sci. 2024 Sep 20;25(18):10129. doi: 10.3390/ijms251810129 (PMC11431867; doi:10.3390/ijms251810129)
Supplement: Supplementary file 1 [file ijms-25-10129-s001.zip › ijms-3183621-supplementary.pdf]

Supplementary information to:

Chromate affects gene expression and DNA methylation in long-term *in vitro* experiments using A549 cells

Franziska Fischer[1], Sandra Stößer[1], Lisa Wegmann[1], Eva Veh[1], Tatjana Lumpp[1], Marlene Parsdorfer[1], Paul Schumacher[1] and Andrea Hartwig[1*]

[1]Department of Food Chemistry and Toxicology, Institute of Applied Biosciences (IAB), Karlsruhe Institute of Technology (KIT), 76131 Karlsruhe, Germany.

* Correspondence: andrea.hartwig@kit.edu

Supplementary Table S1: Genes and the respective encoded proteins analyzed in the present study. Genes marked with * are reference genes.

Gene	Encoded protein
ACTB*	β-actin (ACTB)
APAF1	apoptotic protease activating factor (APAF1)
ATM	ataxia telangiectasia mutated (ATM)
ATR	ataxia telangiectasia and Rad3-related protein (ATR)
B2M*	beta-2-Microglobulin (B2M)
BAX	bcl2-associated x protein (BAX)
BRCA1	breast cancer 1, early onset (BRCA1)
BRCA2	breast cancer 2, early onset (BRCA2)
BTRC	transducin repeat containing E3 ubiquitin protein ligase, beta (β-TrCP)
CAT	catalase (CAT)
CCL22	C-C motif chemokine ligand 22 (CCl22)
CCND1	cyclin D1 (CCND1)
CDKN1A	cyclin-dependent kinase inhibitor 1A (p21)
CDKN1B	cyclin-dependent kinase inhibitor 1B (p27)
CDKN2A	cyclin-dependent kinase inhibitor 2A (p16)
CDKN2B	cyclin-dependent kinase inhibitor 2B (p15)
CDKN2D	cyclin-dependent kinase inhibitor 2D (p19)
COX2	cytochrome C oxidase assembly factor (COX2)
DDB2	damage-specific DNA binding protein 2 (DDB2)
DDIT3	DNA damage inducible transcript 3 (DDIT3)
DNMT1	DNA methyltransferase 1 (DNMT1)
DNMT3A	DNA methyltransferase 3A (DNMT3A)
DNMT3B	DNA methyltransferase 3B (DNMT3B)
E2F1	E2F transcription factor 1 (E2F1)
EGFR	epidermal growth factor receptor (EGFR)

EHMT2/G9a	euchromatic histone lysine methyltransferase 2(EHMT2)
EP300	E1A binding protein P300 (EP300)
ERCC2	excision repair cross-complementation group 2 (XPD)
ERCC4	excision repair cross-complementation group 4 (XPF)
ERCC5	excision repair cross-complementation group 5 (XPG)
FOXO1	forkhead box O1 (FOXO1)
FOXO3	forkhead box O3 (FOXO3)
FTH1	ferritin heavy chain 1 (FTH1)
G6PD	glucose-6-phosphate dehydrogenase (G6PD)
GADD45A	growth arrest and DNA-damage-inducible, alpha (GADD45A)
*GAPDH**	glyceraldehyde-3-phosphate dehydrogenase (GAPDH)
GCLC	glutamate-cysteine ligase, catalytic subunit (GCL)
GPX1	glutathione peroxidase 1 (GPX1)
GPX2	glutathione peroxidase 2 (GPX2)
GSR	glutathione reductase (GSR)
*GUSB**	glucuronidase, beta (GUSB)
HDAC1	histone deacetylase 1 (HDAC1)
HDAC10	histone deacetylase 10 (HDAC10)
HDAC2	histone deacetylase 2 (HDAC2)
HDAC3	histone deacetylase 3 (HDAC3)
HMOX1	heme oxygenase (decycling) 1 (hMO-1)
*HPRT1**	hypoxanthine phosphoribosyltransferase 1 (HPRT1)
HSPA1A	heat shock 70kDa protein 1A (HSP70)
IL1a	interleukin 1 alpha (IL1a)
IL1b	interleukin 1 beta (IL1b)
IL6	interleukin 6 (IL6)
IL8	interleukin 8 (IL8)
JUN	jun proto-oncogene (c-JUN)
KDM3A	lysine demethylase 3A (KDM3A)
KEAP1	kelch-like ECH-associated protein 1 (KEAP1)
LIG1	ligase I, DNA, ATP-dependent (LIG1)
LIG3	ligase III, DNA, ATP-dependent (LIG3)
MAP3K5	mitogen-activated protein kinase kinase kinase 5 (MAP3K5/ASK1)
MBD4	methyl-CpG binding domain 4 (MBD4)
MDM2	MDM2 Proto-Oncogene (MDM2)
MeCP2	methyl-CpG binding protein 2 (MeCP2)
MGMT	O-6-methylguanine-DNA methyltransferase (MGMT)
MLH1	mutL homolog 1 (MLH1)
MSH2	mutS homolog 2 (MSH2)
MT1X	metallothionein 1X (MT1X)
MT2A	metallothionein 2A (MT2A)
MYC	v-myc avian myelocytomatosis viral oncogene homolog (c-MYC)
NFKB1	nuclear factor of kappa light polypeptide gene enhancer in B-cells 1 (p50/p105)
NFKB2	nuclear factor of kappa light polypeptide gene enhancer in B-cells 2 (p49/p100)
NFKBIA	nuclear factor of kappa light polypeptide gene enhancer in B-cells inhibitor, alpha (IKBA)
OGG1	8-oxoguanine DNA glycosylase (hOGG1)

PARP1	poly (ADP-ribose) polymerase 1 (PARP1)
PLK3	polo-like kinase 3 (PLK3)
PMAIP1	phorbol-12-myristate-13-acetate-induced protein 1 (NOXA)
PRDX1	peroxiredoxin 1 (PRX1)
RAD50	RAD50 homolog (S. cerevisiae) (RAD50)
RAD51	RAD51 recombinase (RAD51)
RRM2B	ribonucleotide reductase M2B (TP53 inducible) (p53R2)
SETD2	SET Domain Containing 2 (SETD2)
SIRT2	sirtuin 2 (SIRT2)
SLC30A1	solute carrier family 30 (zinc transporter), member 1 (ZnT1)
SOD1	superoxide dismutase 1, soluble (SOD1)
SOD2	superoxide dismutase 2, mitochondrial (SOD2/MnSOD)
TET1	tet methylcytosine Dioxygenase 1 (TET1)
TET2	tet methylcytosine Dioxygenase 2 (TET2)
TET3	tet methylcytosine Dioxygenase 3 (TET3)
TGFb	transforming growth factor beta (TGFb)
TNFa	tumor necrosis factor a (TNFa)
TNFRSF10B	tumor necrosis factor receptor superfamily, member 10b (DR5)
TXN	thioredoxin (TXN)
TXNRD1	thioredoxin reductase 1 (TXNRD)
VEGFA	vascular endothelial growth factor A (VEGFA)
XPA	xeroderma pigmentosum, complementation group A (XPA)
XPC	xeroderma pigmentosum, complementation group C (XPC)
XRCC5	X-ray repair complementing defective repair in Chinese hamster cells 5 (XRCC5)

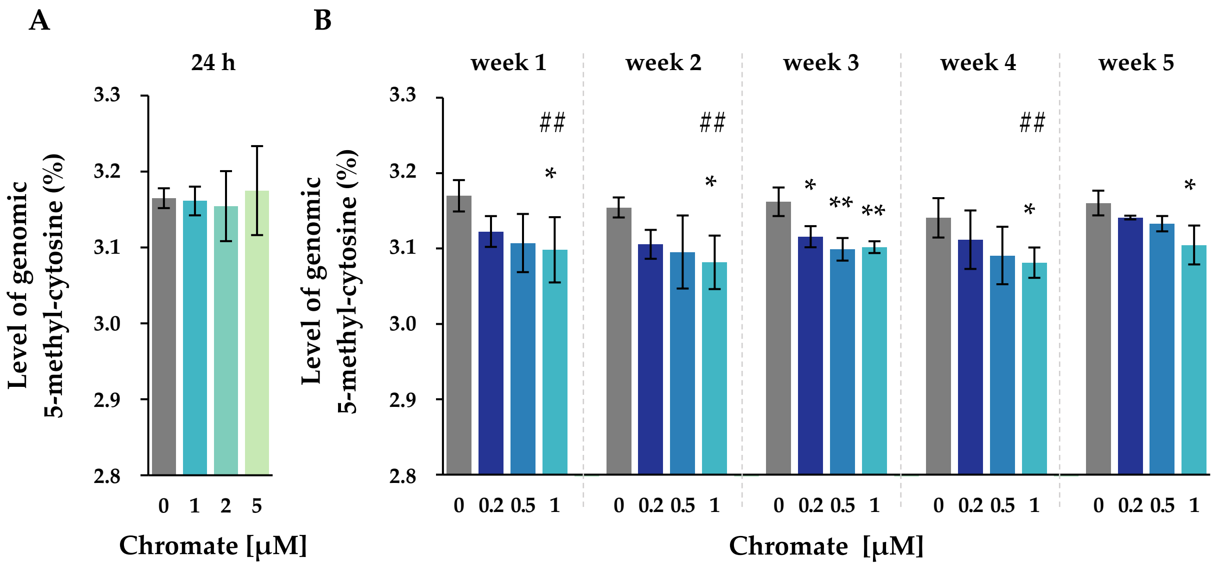

Figure S1: Impact of chromate on the global 5-methyl-cytosine content in A549 cells after 24 hours of treatment (A) and after incubation for 1-5 weeks (B), shown as genomic 5 methyl-cytosine level related to the cytosine content. DNA isolated from untreated or treated cells was digested into single nucleosides and separated by HPLC on a C18 column. Cytidine was detected at 272 nm and 5-methylcytidine at 280 nm. Shown are mean values of three independent experiments per-formed in duplicates ± SD. Statistical analysis was performed to assess differences between treated and untreated cells within a time point using ANOVA followed by Dunnett's T post hoc test. *$p \leq 0.05$, **$p \leq 0.01$. Differences between 1 µM chromate of short-term and long-term treatment was determined by ANOVA followed by Dunnett's T post hoc test. ##: $p \leq 0.01$.

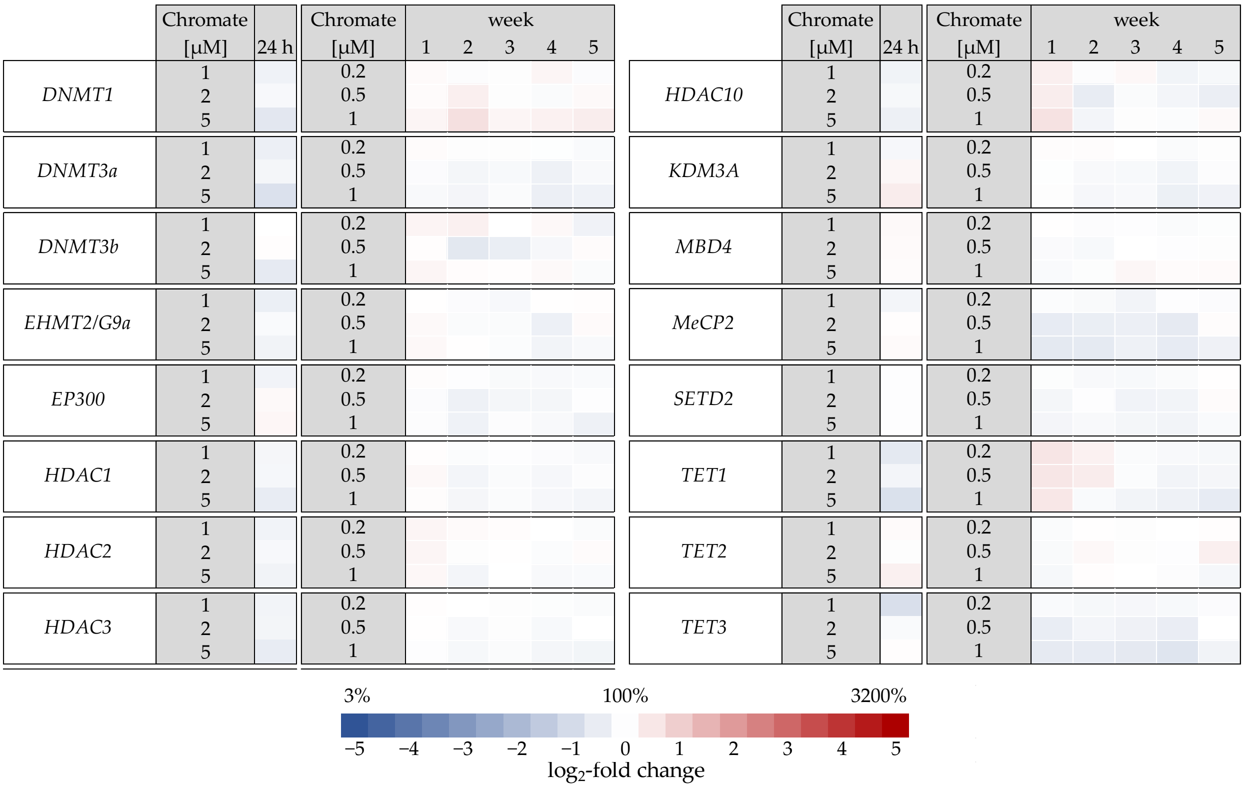

Figure S2: Gene expression profiles of A549 cells treated with chromate for 24 h or 1 to 5 weeks using a high-throughput RT-qPCR. Genes shown encode proteins involved in epigenetic regulation. The log$_2$-changes related to the untreated control are illustrated. Blue color represents a repression; red color represents an induction. Shown are mean values of at least three independent experiments performed in duplicates.

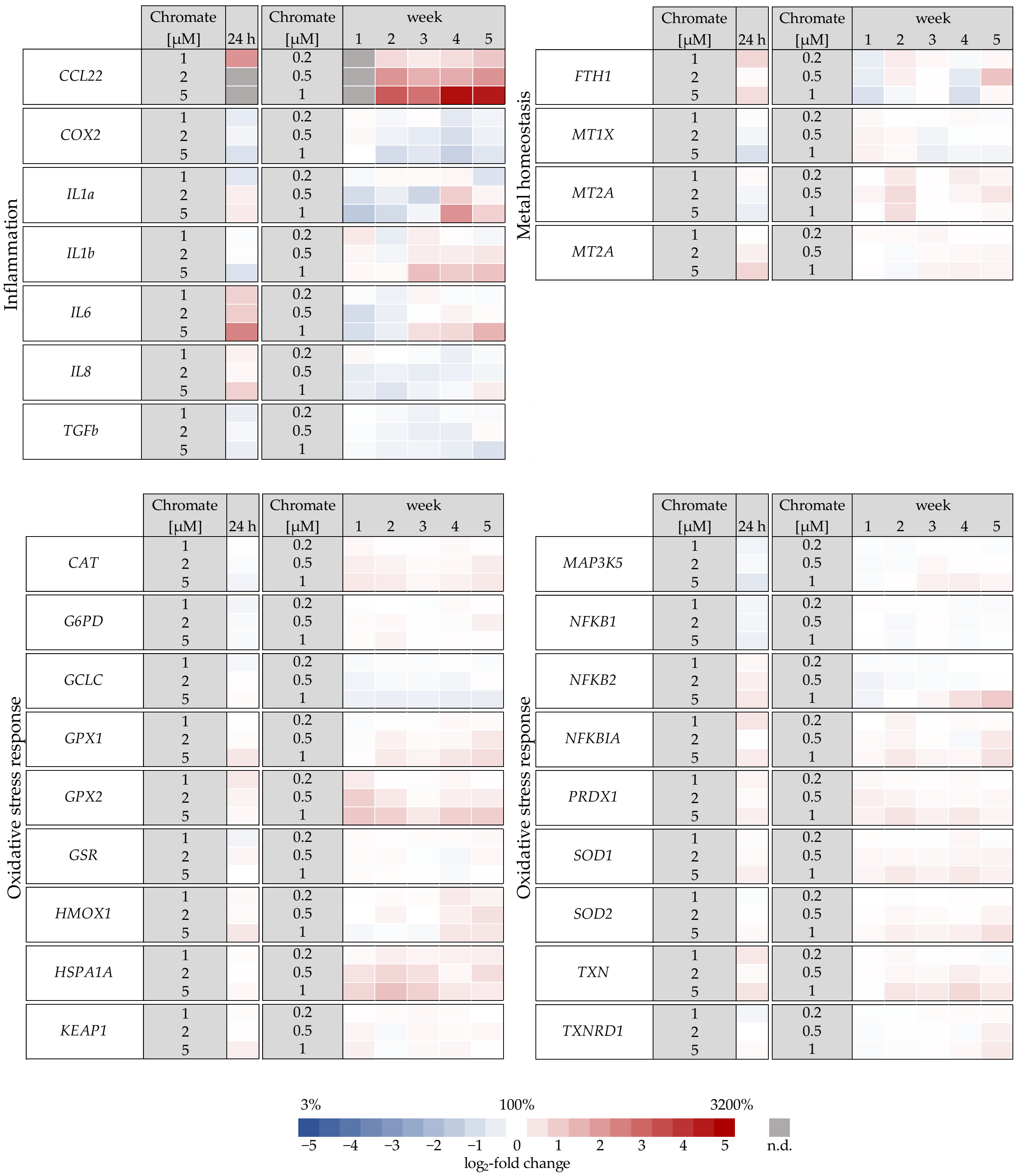

Figure S3: Gene expression profiles of A549 cells treated with chromate for 24 h or 1 to 5 weeks using a high-throughput RT-qPCR. Genes shown encode proteins involved in inflammation, metal homeostasis and oxidative stress response. The log_2-changes related to the untreated control are illustrated. Blue color represents a repression; red color represents an induction. Shown are mean values of at least three independent experiments performed in duplicates.

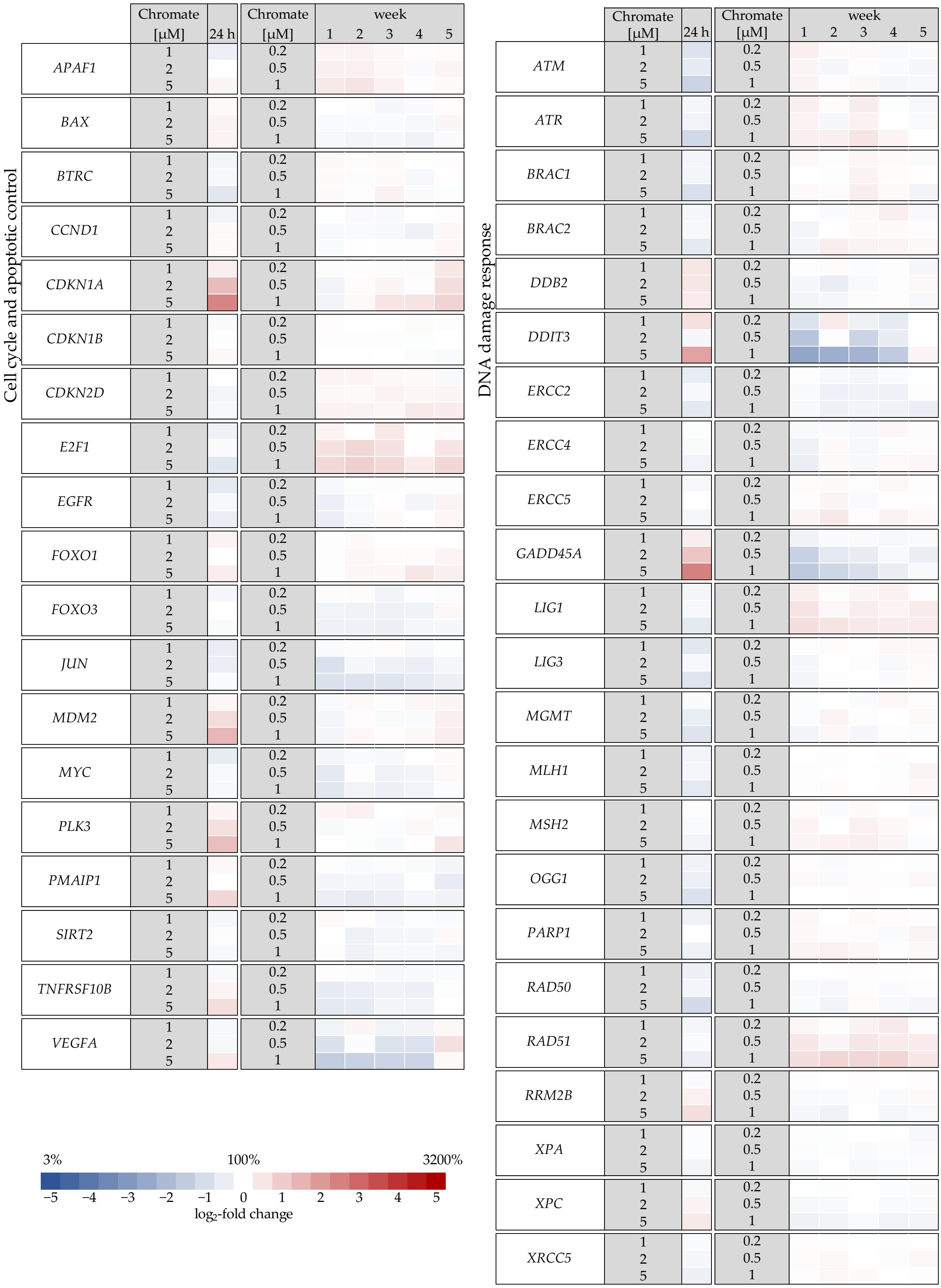

Figure S4: Gene expression profiles of A549 cells treated with chromate for 24 h or 1 to 5 weeks using a high-throughput RT-qPCR. Genes shown encode proteins involved in cell cycle and apoptotic response as well as DNA damage response. The $\log_2$-changes related to the untreated control are illustrated. Blue color represents a repression; red color represents an induction. Shown are mean values of at least three independent experiments performed in duplicates.
